# Clinic Notes from the McKillip Veterinary College

**Published:** 1898-07

**Authors:** L. A. Merillat


### CLINIC NOTES FROM McKILLIP VETERINARY COLLEGE.

Reported by Dr. L. A. Merillat.

Tracheal Stenosis. The possibility of successfully replacing a considerable portion of a horse's trachea with an artificial tube was demonstrated at a surgical clinic January 7, 1898.

Following two operations of tracheotomy, the patient's trachea collapsed so as to lessen its lumen to a dangerous extent. The subsequent operation showed that seven rings had been cut. The integument and muscles were dissected from the trachea and all the cut rings removed, except a small part of their dorsal portion. An aluminum tube, 17.5 centimetres (7 inches) in length and of equal calibre to that of the normal trachea, was sutured with silver wire to the rings at each extremity of the wound. The muscles and integument were then carefully brought together over it. Union took place in due time, and the animal, six months later, shows little or no inconvenience and works daily.

This case is of special importance, as tracheal stenosis following even simple tracheotomy is quite common, and as, in most cases, only two or three rings are involved, the operation would ordinarily be attended with even less danger and inconvenience to the animal.

---

George Wilkes's bones have been unearthed after sixteen years' burial, and presented to the Museum of the Kentucky State College at Lexington, a fitting place for the skeleton of so prepotent a sire.

German canaries excel all other canaries as singers. A canary of Germany has been known to continue a single trill for a minute and a quarter, with twenty changes of note in it.

Cows that are wild and unruly may be led without difficulty by blindfolding them.

Blue-ribbon horses in certain classes will be excluded from competition at the next exhibition of the National Horse-show, Madison Square Garden, New York.

Another shoe with removable calks for asphalt pavements—the calk on the wearing surface to be circular or tubular in form.

The average bullock weighs 800 pounds.

